# Imaging cardio-obstetrics: a multidisciplinary model of care

**DOI:** 10.1093/ehjimp/qyaf083

**Published:** 2025-07-10

**Authors:** Monica Baroni, Pierluigi Festa, Francesca Chesi, Sabrina Costa

**Affiliations:** Cardiothoracic Department, Fondazione Monasterio Pisa – Massa, Via Moruzzi 1 Pisa 56127, Italy; Pediatric Department, Fondazione Monasterio – Ospedale del Cuore, Massa, Italy; Pediatric Department, Fondazione Monasterio – Ospedale del Cuore, Massa, Italy; Pediatric Department, Fondazione Monasterio – Ospedale del Cuore, Massa, Italy

The rise of cardio-obstetrics as a dedicated specialty reflects a growing awareness that pregnancy, while physiological, represents a period of significant cardiovascular stress. As recently outlined,^1^ cardiovascular disease (CVD) has emerged as a leading cause of maternal mortality in high-income countries, often with preventable outcomes. Moreover, pregnancies are increasingly occurring in women of advanced maternal age, many of whom may have pre-existing comorbidities, including congenital or acquired cardiovascular conditions. This evolving landscape requires structured, multidisciplinary, and technologically integrated models of care.

The Fondazione Monasterio in Tuscany, Italy, has developed a unique model of cardio-obstetric care within its hospital in Massa, historically dedicated to paediatric cardiology and congenital heart disease. This model is integrated with regional healthcare networks, allowing for referrals from other hospitals and healthcare facilities across Tuscany. At Monasterio, cardiologists, obstetricians, anaesthesiologists, neonatologists, imaging specialists, psychologists, geneticists, and clinical bioengineers collaborate seamlessly from preconception to postpartum, ensuring tailored, dynamic, and safe care for both mother and baby.

Our cardio-obstetric pathways include the multidisciplinary evaluation of women with congenital or acquired heart disease—including those with complex grown-up congenital heart disease—who seek counselling on the feasibility and safety of pregnancy. Risk stratification is performed according to current international guidelines, with shared decision-making guiding reproductive choices and care planning. In addition, we serve as a regional hub for the prenatal diagnosis and management of foetal cardiac pathology, ensuring early detection and coordinated perinatal care across the Tuscany region (*Figure 1*).

Pregnant patients with CVD are followed throughout gestation by an integrated team that combines advanced imaging (echocardiography, cardiac magnetic resonance imaging, and computed tomography when appropriate) with data-driven tools provided by clinical bioengineering. Where needed, foetal and maternal cardiovascular anatomy is reconstructed using 3D modelling techniques, enabling pre-procedural simulation and surgical planning in the case of complex congenital heart defects.

This integration of cardiovascular imaging and bioengineering not only supports diagnosis and risk stratification but also enables dynamic planning of delivery and peripartum management, especially in high-risk conditions such as peripartum cardiomyopathy, aortic disease, or severe valvular pathology. Imaging plays a central role in our multidisciplinary board discussions and perinatal strategy meetings, enhancing both safety and coordination among specialties.

A key feature of our centre is the presence of a dedicated, integrated birthing unit for women with cardiac conditions. Located adjacent to the paediatric cardiac surgery unit and neonatal intensive care, this setting allows for seamless delivery management—whether vaginal or caesarean—and immediate neonatal assessment and intervention in cases of prenatally diagnosed congenital heart defects. Our centre is BFHI-certified, with a strong focus on supporting breastfeeding and skin-to-skin contact even in high-risk contexts. We actively promote these practices for mothers and newborns with cardiac conditions, recognizing their vital role in enhancing bonding, physiological stability, and long-term outcomes. This commitment is particularly relevant in the management of prenatally diagnosed congenital heart disease, where early postnatal care must balance clinical complexity with humanized care. This approach is especially valuable for foetuses with diagnosed congenital heart disease, where early bonding and physiological stability are crucial. While the model was originally designed for maternal cardiac disease, it is now equally and powerfully applied to pregnancies complicated by foetal cardiac pathology, following the same integrated care pathway.

Newborns diagnosed with congenital heart disease through prenatal imaging are taken into care by a single continuum of professionals—from foetal diagnosis to postnatal intervention. This includes interventional cardiologists, cardiac surgeons, and neonatologists, working in close connection with the maternal care team to optimize outcomes for both patients.

Monasterio’s model also includes research and education as pillars of cardio-obstetric development. Clinical pathways are constantly refined based on outcomes analysis and integration of new technologies, and our training programs are designed to expose both cardiologists and obstetricians to the complexities of managing CVD in pregnancy.

By embedding imaging and bioengineering into every stage of care, from pre-pregnancy counselling to postpartum follow-up, Monasterio provides a concrete example of how cardio-obstetrics can evolve from a collaboration into a true specialty—with dedicated processes, tools, and professional cultures.

This model could inspire similar hubs elsewhere, as the field moves toward structured accreditation, interdisciplinary training, and increased research. Ultimately, ensuring that women with heart disease can embark on motherhood safely is not only a clinical mission—it is a matter of equity, dignity, and innovation.

**Figure 1:**
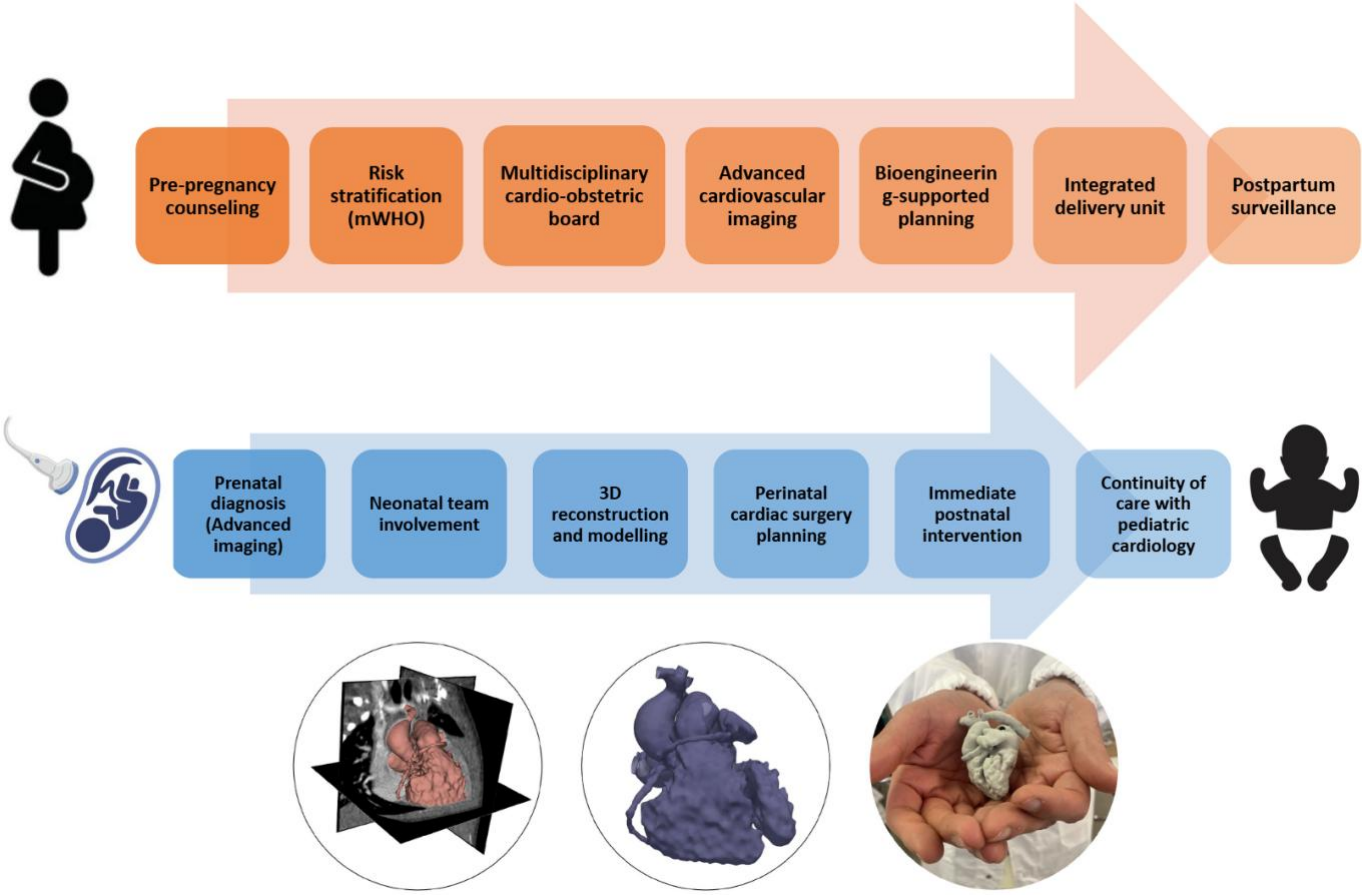
The figure represents the clinical and imaging pathway for pregnant women with a diagnosis of foetus cardiac disease and/or for women with cardiac disease who want to become pregnant, to guarantee appropriate and safe management of pregnancy and birth.

## Data Availability

No new data were generated or analysed in support of this research.

## Lead author biography

Monica Baroni, MD, is an expert in clinical cardiology with special focus on echocardiography and multimodality imaging. Sha has extensive experience in clinical pathway management and clinical governance at the Fondazione Monasterio.
